# Application of a single-objective, hybrid genetic algorithm approach to pharmacokinetic model building

**DOI:** 10.1007/s10928-012-9258-0

**Published:** 2012-07-06

**Authors:** Eric A. Sherer, Mark E. Sale, Bruce G. Pollock, Chandra P. Belani, Merrill J. Egorin, Percy S. Ivy, Jeffrey A. Lieberman, Stephen B. Manuck, Stephen R. Marder, Matthew F. Muldoon, Howard I. Scher, David B. Solit, Robert R. Bies

**Affiliations:** 1 Division of Clinical Pharmacology, Department of Medicine, Indiana University School of Medicine, Indianapolis, IN USA; 2 Roudebush Veterans Affairs Medical Center, Indianapolis, IN USA; 3 Next Level Solutions, Raleigh, NC USA; 4 Centre for Addition and Mental Health, University of Toronto, Toronto, ON Canada; 5 Rotman Research Institute, Baycrest Hospital, University of Toronto, Toronto, ON Canada; 6 Department of Psychiatry, School of Medicine, University of Pittsburgh, Pittsburgh, PA USA; 7 Penn State Hershey Cancer Institute, Hershey, PA USA; 8 University of Pittsburgh Cancer Institute, Pittsburgh, PA USA; 9 National Cancer Institute, Bethesda, MD USA; 10 Department of Psychiatry, Columbia University College of Physicians and Surgeons, New York, NY USA; 11 Behavioral Physiology Laboratory, Department of Psychology, University of Pittsburgh, Pittsburgh, PA USA; 12 Department of Psychiatry and Biobehavioral Sciences, University of California at Los Angeles School of Medicine, Los Angeles, CA USA; 13 Center for Clinical Pharmacology, University of Pittsburgh, Pittsburgh, PA USA; 14 Sidney Kimmel Center for Prostate and Urologic Cancers, Memorial Sloan-Kettering Cancer Center, New York, USA; 15 Department of Medicine, Memorial Sloan-Kettering Cancer Center, New York, NY USA

**Keywords:** Pharmacokinetics, Model building, Genetic algorithms

## Abstract

A limitation in traditional stepwise population pharmacokinetic model building is the difficulty in handling interactions between model components. To address this issue, a method was previously introduced which couples NONMEM parameter estimation and model fitness evaluation to a single-objective, hybrid genetic algorithm for global optimization of the model structure. In this study, the generalizability of this approach for pharmacokinetic model building is evaluated by comparing (1) correct and spurious covariate relationships in a simulated dataset resulting from automated stepwise covariate modeling, Lasso methods, and single-objective hybrid genetic algorithm approaches to covariate identification and (2) information criteria values, model structures, convergence, and model parameter values resulting from manual stepwise versus single-objective, hybrid genetic algorithm approaches to model building for seven compounds. Both manual stepwise and single-objective, hybrid genetic algorithm approaches to model building were applied, blinded to the results of the other approach, for selection of the compartment structure as well as inclusion and model form of inter-individual and inter-occasion variability, residual error, and covariates from a common set of model options. For the simulated dataset, stepwise covariate modeling identified three of four true covariates and two spurious covariates; Lasso identified two of four true and 0 spurious covariates; and the single-objective, hybrid genetic algorithm identified three of four true covariates and one spurious covariate. For the clinical datasets, the Akaike information criterion was a median of 22.3 points lower (range of 470.5 point decrease to 0.1 point decrease) for the best single-objective hybrid genetic-algorithm candidate model versus the final manual stepwise model: the Akaike information criterion was lower by greater than 10 points for four compounds and differed by less than 10 points for three compounds. The root mean squared error and absolute mean prediction error of the best single-objective hybrid genetic algorithm candidates were a median of 0.2 points higher (range of 38.9 point decrease to 27.3 point increase) and 0.02 points lower (range of 0.98 point decrease to 0.74 point increase), respectively, than that of the final stepwise models. In addition, the best single-objective, hybrid genetic algorithm candidate models had successful convergence and covariance steps for each compound, used the same compartment structure as the manual stepwise approach for 6 of 7 (86 %) compounds, and identified 54 % (7 of 13) of covariates included by the manual stepwise approach and 16 covariate relationships not included by manual stepwise models. The model parameter values between the final manual stepwise and best single-objective, hybrid genetic algorithm models differed by a median of 26.7 % (*q*_1_ = 4.9 % and *q*_3_ = 57.1 %). Finally, the single-objective, hybrid genetic algorithm approach was able to identify models capable of estimating absorption rate parameters for four compounds that the manual stepwise approach did not identify. The single-objective, hybrid genetic algorithm represents a general pharmacokinetic model building methodology whose ability to rapidly search the feasible solution space leads to nearly equivalent or superior model fits to pharmacokinetic data.

**Electronic supplementary material:**

The online version of this article (doi:10.1007/s10928-012-9258-0) contains supplementary material, which is available to authorized users.

## Introduction and background

Two key objectives in population pharmacokinetic model building, removing systematic errors (bias) and minimizing the amount of unexplained variance between the experimental data and the model predictions, are accomplished by selecting an appropriate model structure, identifying fixed and random effects, and including characteristics of individuals (covariates) in the model. Decisions regarding specifics of the model structure are commonly made in series to isolate a potential model component (e.g., additional compartment, covariate relationship) and evaluate the effect, an algorithm referred to as stepwise regression [1]. For example, in the stepwise forward/backward approach to model building, the model structure (e.g., number of compartments, presence of an absorption lag time, etc.) is selected, the inter-individual variability and residual error structures are chosen, and then the effect of either adding an individual covariate to the base model (forward addition) or removing an individual covariate from a model which includes all included covariates (backward elimination) is evaluated. However, covariates may become relevant (or irrelevant) in combination and the inclusion of covariates and error terms can affect the fit characteristics of the compartment model [2]. Such higher order interactions are often difficult to identify because the number of possible combinations of model decisions is prohibitive (although skilled pharmacokinetic modelers may be able to identify some higher order interactions through diagnostic plots, personal experiences, or understanding of the biology and pharmacology) without the use of combinatoric optimization techniques. Bies et al. [3] discussed the suitability of various multi-variable optimization methods to pharmacokinetic model covariate and structure decision making before selecting a single-objective, hybrid genetic algorithm (SOHGA) based modeling building method, coupling the SOHGA to NONMEM for parameter estimation, and demonstrating its application on pharmacokinetic data for a single compound. In the paper by Bies et al. [3], the SOHGA resulted in a best-fit candidate model with the same compartmental structure as the stepwise method but with a statistically better fit to the data, additional covariates, and lower between patient variability. In this manuscript the use of SOHGA for pharmacokinetic model building is evaluated by comparing the selection of covariates and model parameter estimates using automated stepwise covariate selection, Lasso, and SOHGA for a simulated dataset and fits to plasma concentration data for the final models selected by these manual stepwise versus SOHGA approaches for each of seven, prospectively identified, compounds. In addition, we compare the final model structure, completion of model convergence and covariance steps, inclusion of inter-individual and inter-occasion variability, covariates included, and percent difference in the pharmacokinetic model parameters between the two model building approaches to clinical data.

### Genetic algorithms

Genetic algorithms are a class of global search techniques commonly used in engineering and science for multi-variable parameter estimation; this tool has been successfully applied to problems such as to electrical distribution [4], biological networks [5], and scheduling processes [6]. Perhaps more remarkably, and relevant to this application, a genetic algorithm method was able to derive laws of nature without any prior knowledge of physics or geometry, based solely on observed data [7]. The name “genetic algorithm” refers to the method’s operators which mimic the processes of biological natural selection. A basic genetic algorithm maintains a finite pool of candidate models which “evolve” from generation to generation by applying operators that “mate” and “mutate” candidate models of the current generation to form a new generation of candidate models (see Goldberg [8] for details on genetic algorithm implementation). These mating, or cross-over, and mutation operators require that models are represented in binary notation where bit strings coding for individual model features are concatenated to form a model string. In the cross-over operator, two “parent” models are selected and the first *N* number of bits swapped to form two offspring models where *N* is a uniform random distribution of the number of bits in the genome string. The mate selection is not an entirely random as most genetic algorithms weight a candidate model’s mating likelihood by the model’s performance. This implies that candidate models with advantageous qualities (e.g., low value for −2 × log likelihood, more parsimonious, successful convergence, etc.) are more likely to be involved in mating, those qualities are more likely to be seen, perhaps multiple times, in the next generation of candidate models, and candidate models in subsequent generations tend to have better performance. The mutation operator randomly changes a binary bit within a candidate model which can lead to further exploration of the solution space and advanced techniques such as niching and elitism keep the candidates from collapsing into a single region and keep the best performing candidate models intact between generations, respectively. Although convergence to the global optimum is not guaranteed, properly applied genetic algorithms have been shown to be quite robust, with only very irregular and complex solution spaces being “GA deceptive” [8].

### Single-objective genetic algorithms

The “single-objective” approach uses a single outcome as a measure of a candidate model’s fitness. While this fitness can be based on a single objective, frequently multiple objectives (e.g., −2 × log likelihood, parsimony, convergence, covariance, correlation, eigenvalues, etc.) are combined into a single, composite outcome measure using user defined weights. New approaches can be used to simultaneously optimize on multiple objectives [9].

### Hybrid genetic algorithms

While simple genetic algorithms are efficient at identifying “good” regions of the solution space, they are inefficient at identifying a locally optimal solution within a “good” region of the search space. This is because, although the cross over operator is effective at identifying generally good regions of solution space, identifying the local optima is limited by the rate of mutation, a relatively low frequency event. As such, a hybrid approach is one that complements the globally-oriented genetic algorithm with a local search method to refine candidate model selection.

For example, in Bies et al. [3] a simple genetic algorithm was interrupted every fifth generation by a simple downhill local search method. In the simple downhill search, the fitness of a candidate model is compared with all models whose strings differ by a single bit and the best model replaces the original candidate model as a candidate model. This process is repeated until no further improvement is seen. At this point, the genetic algorithm is resumed, with the new best candidate models.

### Application of a single-objective, hybrid genetic-algorithm to pharmacokinetic model building

Bies et al. [3] applied the SOHGA framework to pharmacokinetic model building by mapping pharmacokinetic model decision outcomes to binary strings in such a way that the binary string for each outcome of a given decision is unique. The strings corresponding to individual model decisions (e.g. 1 vs. 2 compartments, with or without lag time, initial estimate for Volume of 10 vs. 100 L) are concatenated to form the model string whose particular binary values define a candidate model structure. That is, each model decision is contained in a binary string segment with a conserved initiation and termination location within model strings. The binary pattern uniquely specifies the outcome for that model decision and the collection of all model decision outcomes for a particular model string specifies a candidate model. The SOHGA methodology was then applied by building a set of candidate models, running each in NONMEM to estimate the model parameters, describing the fitness of candidate models with a single objective function value, and performing hybrid, genetic algorithm operations.

## Methods

We took two approaches to assess the application of SOHGA for pharmacokinetic model selection. The first approach was to fit pharmacokinetic models to longitudinal drug concentration data simulated using known population parameters and covariate effects. The second approach was to fit pharmacokinetic models to longitudinal drug concentration data from clinical trials for each of seven compounds: intravenous administration of citalopram and 17-(dimethylaminoethylamio)-17-demethoxygeldanamycin (DMAG) and oral dosing of escitalopram, olanzapine, perphenazine, risperidone, and ziprasidone.

For the simulated dataset, only covariate identification was tested. That is, the compartment structure, inter-individual and residual error structures were fixed as the “true” model. Three automated modeling building approaches were implemented: stepwise covariate modeling (SCM) [10], Lasso [11], and SOHGA. We tested three automated SCM forward inclusion/backward elimination *p* value criteria (0.05, 0.05; 0.05, 0.01; and 0.10, 0.01) and two SOHGA methods with 3.84 and 10 point penalties per parameter. Each model approach was evaluated based on “true” and spurious covariates identified and the objective function value.

To build the pharmacokinetic models for the clinical compounds, two different model building approaches were tested: a manual method (implemented by an experienced NONMEM user) using stepwise forward inclusion/backward elimination for covariate selection and a single-objective, hybrid genetic algorithm. Both modeling approaches made decisions on covariate inclusion as well as functional forms of the covariate relationships, inter-individual variability, and residual error. The quality of best fitting models identified using the stepwise and hybrid genetic algorithm model development approaches were then compared for each compound. In addition, the covariates included, the precision of model parameters, and the completeness of the searches are reported. Each modeling approach was performed blinded to the results of the other approach.

### Simulated dataset

The simulated data was created with two objectives in mind: (1) a highly identifiable model and (2) realistically high correlations between covariates. Data were simulated for 200 subjects given a single, intravenous dose using the ADVAN1/TRANS2 subroutine in NONMEM 7.2. Sample times were 0.25, 0.5, 1, 2, 3, 6, 10, and 24 h after the dose.

We created realistically correlated covariates (Table 1). First, the uncorrelated covariates were simulated. These were body-mass index (BMI), sex, creatinine (CR), and age. Next, dependent covariates were simulated. These were height (HT) as a function of sex, weight (WT) as a function of body-mass index and height, body-surface area (BSA) as a function of height and weight [12], and creatinine clearance (CRCL) as a function of weight, age, creatinine, and sex [13]. In addition to the covariates described above that directly or indirectly entered into the model, four unrelated or “spurious” covariates (CV1 to CV4) were included. The correlation matrix for the simulated covariates and NONMEM code for generating the simulated dataset is given in Appendix A.

**Table 1:** Distribution characteristics of covariates used for simulated dataset

Covariate	Source	Mean, expected (observed)	Standard deviation, expected (observed)
Body mass index (BMI)	Log normal	26.0 kg/m^2^ (26.3)	0.15 kg/m^2^ (0.156)
Sex (SEX)	Discreet (0,1)	–	0.5 males, 0.5 females
			(0.47 males, 0.53 females)
Age (AGE)	Normal	40 years (38.8 years)	8 years (8.18 years)
Creatinine (CR)	Log normal	1.0 mg/Dl (0.98 mg/Dl)	0.13 mg/DI (0.12 mg/DI)
Height (HT)	Mean of 1.7 for males, 1.5 for females	1.6 m (1.59 m)	0.2 m
Weight^a^ (WT)	BMI × HT^2^	67.4 kg	21.2 kg
Body-surface area^a^ (BSA)	$\sqrt {{\text{HT}} \times 100 \times {\text{WT}}/3600}$	1.71 m^2^	0.363 m^2^
Creatinine clearance^a^ (CRCL)	(140 × AGE) × WT/(72 × CR)	98 mL/min	36 mL/min
Unrelated covariate 1 (CV1)	Log normal	100 (97.3)	0.3 (0.31)
Unrelated covariate 2 (CV2)	Log normal	10 (10.1)	0.4 (0.42)
Unrelated covariate 3 (CV3)	Log normal	1.0 (0.99)	0.2 (0.21)
Unrelated covariate 4 (CV4)	Log normal	0.1 (0.096)	0.2 (0.21)

^a^There is no expected mean or expected standard deviation for calculated covariates

The simulated data contained exponential effects of body-mass index and creatinine clearance on clearance, exponential effects of body-surface area and sex on volume of distribution, lognormal inter-individual effects on both clearance and volume of distribution, and a combined residual error structure. The magnitude of the covariate effect was selected to provide a range of effects on the NONMEM objective function values (between 10 and 100 points for each covariate inclusion) seen in analysis of real data.

The clearance and volume of distribution were set to generate data over a 24 h sampling period that would permit their estimation. The baseline clearance was 0.763 L/h and the baseline volume of distribution was 1.94 L. These baseline values give an expected half-life of 1.76 h. This resulted in data for at least 3 elimination half-lives for the vast majority of simulated subjects.

### Clinical datasets

The study was approved by the Indiana University Institutional Review Board. The study protocols for data collection were approved by the Institutional Review Boards at the respective institutions and all patients, or his or her legal guardian, provided informed consent.

Individuals who received oral olanzapine [14], perphaenazine [15], risperidone [16], or ziprasidone [17] were patients treated between January 2001 and December 2004 as part of the Clinical Antipsychotic Trials of Intervention Effectiveness (CATIE)—a multisite, US study of randomized, double-blind, medication comparative-effectiveness trial—in the treatment of Alzheimer’s (CATIE-AD) and schizophrenia (CATIE-SZ). Details of the inclusion, exclusion, and study design can be found in Stroup et al. [18] for the schizophrenia studies and Schneider et al. [19] for Alzheimer’s studies with a summary of the treatment protocol and final enrollment statistics shown in Table 2. Individuals who received oral escitalopram were patients treated for major depression as part of the clinical trial “Depression: The Search for Treatment Relevant Phenotypes” [20, 21]. Individuals who received intravenous citalopram were part of a cross sectional study of healthy volunteers participating in Pittsburgh’s Adult and Human Behavior Project; these individuals were between the ages of 30 and 55 years and not taking medications for hypertension, lipid disorders, or diabetes [22]. Individuals who received intravenous DMAG were cancer patients either at Pittsburgh’s Cancer Institute or at Memorial Sloan-Kettering participating in a phase II study.

**Table 2:** Sample sizes and treatment protocols

Compound	Number of patients	Number of concentration measurements	Treatment protocol
Citalopram, IV [22]	331^a^	1,324	0.33 mg/kg lean body mass infusion over 30 min^b^
DMAG, IV	67 (48 Pittsburgh, 19 MSK)	1,148 (1,000 Pittsburgh, 148 MSK)	Median of 36.0 mg/m^2^ (range from 2.5 to 160.1 mg/m^2^)^c^
Escitalopram, oral [21]	172 (105 Pittsburgh, 67 Pisa)	473 (320 Pittsburgh, 153 Pisa)	5–20 mg/day, 1×/day^d^
Olanzapine, oral [14]	523 (117 AD, 406 SZ)	1,527 (200 AD, 1,327 SZ)	AD: 2.5–20 mg/day, 1×/day^e^
			SZ: 7.5–30 mg/day, 1–2×/day^f^
Perphenazine, oral [15]	156 (all SZ)	421 (all SZ)	AD: Not applicable^e^
			SZ: 8–32 mg/day, 1–2×/day^f^
Risperidone, oral [16]	490 (110 AD, 380 SZ)	1,236 (168 AD, 1,068 SZ)	AD: 0.5–6 mg/day, 1–2×/day^e^
			SZ: 1.5–6 mg/day, 1–2×/day^f^
Ziprasidone, oral [17]	233 (all SZ)	568	AD: Not applicable^e^
			SZ: 40–160 mg/day^f^

^a^Three patients excluded from analysis by Muldoon et al. [18] (two patients because of missing citalopram measurements and one patient with an outlier prolactin response) were retained in the current dataset

^b^Blood samples for citalopram concentration measurement were drawn at 30, 45, 90, and 150 min [18]

^c^Patients at Memorial Sloan-Kettering (MSK) received a single infusion and the DMAG concentration was measured at 0, 0.5, 0.93, 1.5, 2, 4, 6, 24, and 48 h after the infusion was started. Patients at the Pittsburgh Cancer Institute received either 3 infusions (at 0, 25, and 49 h) or 5 infusion (with additional infusions at 73 and 97 h) and the DMAG concentration was measured at the start of each infusion as well as 0.5, 0.92, 1.08, 1.17 1.25, 1.5, 2, 3, 5, 13, and 17 h after the start of the first, third, and fifth infusions

^d^Patients were treated for a minimum of 32 weeks with blood samples collected at weeks 4, 12, 24, and 36 [16]

^e^Alzheimer’s (AD) patients were treated for 12 weeks with blood plasma drawn at weeks 2, 4, and 12 as well as when medication was switched. The mediation could be switched at week 2 (and the 12 week treatment period begins anew) if it was ineffective or induced side-effects

^f^Schizophrenia (SZ) patients were treated for 18 months with blood samples collected every three months. Patients with an initial treatment failure were offered a choice between open-label treatment or re-randomization

### Manual stepwise forward inclusion/backward elimination approach for modeling clinical datasets

Manual stepwise model results were taken from the literature [14–17, 21] or were generated from unpublished data (DMAG) or data reported elsewhere in the literature [22]. These models were determined using similar methods. Briefly, for each compound, a covariate free model was used to select the model compartment structure with the combination of inter-individual and residual variability structures resulting in the lowest objective function value calculated by NONMEM (twice the negative log likelihood plus a fixed constant offset) and a reasonable distribution of residuals as the base model.

Univariate evaluations of covariates were then performed. Initially, each covariate was included individually in the base model and individual predicted values were calculated using either first-order (risperidone only) or first-order conditional estimation with interaction using Xpose [23] for all possible covariates and function forms. The covariate and its function form that (1) yielded the lowest objective function conditional on (2) improved the objective function value by at least 3.84 (citalopram, DMAG, and [14, 15, 17, 21]) or 7.88 [16] points compared to the base model (which corresponds to Chi-squared values of *p* = 0.05 and *p* = 0.005 with one degree of freedom, respectively) and (3) in which there appeared to be a graphical relationship between the deviation in an individual predicted value and the covariate value across individual predicted values was included in the model. The remaining covariates were then included individually in the updated model, the inclusion criteria evaluated, and the model sequentially updated with additional covariates until no further covariates met the model inclusion criteria.

Finally, individual covariates were removed from the final model and the change in the objective function was calculated. Covariates were excluded from the final model if, when the covariate was removed, the objective function value increased by less than 3.84 (citalopram, DMAG, [14, 15, 21]), 6.63 [17] (a Chi-squared value of *p* = 0.01 with one degree of freedom), or 7.88 [16]. Although the models were derived using different versions of NONMEM, the final models reported in the literature were re-run in NONMEM VI, Level 1.0 using Fortran G77 and NONMEM VII, version 7.2 with Intel Fortran to confirm that the results were independent of the NONMEM version.

### Single-objective, hybrid genetic algorithm approach

The SOHGA operates on a set of candidate models in which each candidate specifies covariates included the in model, their functional forms, the structure of the inter-individual and residual variability, and the initial pharmacokinetic model parameter estimates [3]. Special-use software in which the SOHGA, coded and run in Microsoft Visual Studio and Compaq Digital Fortran, creates NONMEM control files to fit parameters and calculate the fitness of candidate models using NONMEM VI, Level 1.0 or NONMEM VII, version 7.2 using either first-order (risperidone only) or first-order conditional estimation with interaction using Xpose [23]. The fitness function for a candidate model was its objective function value plus a parsimony penalty of 10 points for each covariate, residual error, and inter-individual error parameter; a 400 unit penalty if the minimization itself or the covariance matrix did not converge; and a 300 unit penalty if any of the absolute values of the off-diagonal elements of the estimation correlation matrix were >0.95. These penalties are outlined in Bies et al. [3] although the 300 unit penalty if the ratio of the largest to smallest eigenvalues, or the condition number, was >1,000 was not implemented because condition numbers were not considered during the manual searches. For compounds in which the best SOHGA candidate had a condition number >1,000, we performed a post hoc SOHGA analysis that included the condition number penalty.

The SOHGA retained a population of 200–400 candidate models for 30–50 generations. A total of 0.7 crossover events were expected per set of parents. Candidate models for crossover were randomly selected based on scaled fitness function values; linear regression was performed using all fitness functions within two standard deviations of the mean, the fitness of the regression values were scaled to between 0.2 and 4, and outlying values were assigned one of these extreme values. This scaling of the fitness function improves convergence by not permitting outliers to dominate the selection process. The mutation rate was 0.01 mutations per bit. The candidate model with best fitness function was retained to the next generation, a technique known as elitism. Niche evaluation (where a niches differ by <4 bits) was performed. In niche evaluation, a penalty is applied to models that are similar to other models (differ by 4 or fewer bits). This prevents early convergence, maintaining diversity in the population. Four niches were used, insuring that at least four regions of the search space were explored in each phase of the search. A simple downhill search was performed every five generations. In the downhill search, each bit of the best model in each niche in reversed (0 to 1, 1 to 0) and the resulting models run. For example, if the bit string if of length 40, the downhill search will generate 40 new models for each niche that each differ by a single bit. If any of the resulting models are better than the current best model for that niche, the process is repeated with that model, until no further improvement is seen. Finally, timeout conditions were set where NONMEM timed out (terminated without completion failing convergence) after up to 600 min for a model and a generation timed out after up to 6,000 min although the exact timeout conditions varied by compound.

### Pharmacokinetic model structures for modeling clinical datasets

Only pharmacokinetic model structures considered in the stepwise approach for modeling a compound were options for the SOHGA approach for that compound (see Table 3). This was done to isolate the search part of the model selection process; there was no intent to reproduce the hypothesis generation part of the process. In addition, in a secondary analysis, the SOHGA approach was applied to the risperidone dataset using FOCE with interaction. This was done to determine whether the SOHGA approach could identify a model that successfully converges for a scenario in which the stepwise approach did not.

**Table 3:** Model structure and covariate options for the stepwise and single-objective, hybrid genetic algorithm model building approaches

Compound	NONMEM model structures tested^a,b^	First-order (FO) or first-order conditional (FOCE) estimation	Covariates analyzed^c^
Citalopram, IV	ADVAN3, TRANS4	FOCE with interaction	Continuous: age, body-mass index, fat mass, fat-free mass, weight
			Discrete: sex
DMAG, IV	ADVAN3, TRANS4	FOCE with interaction	Continuous: age, albumin, alanine aminotransferase, aspartate transaminase, bilirubin, blood urea nitrogen, body surface area, creatinine, weight
	ADVAN11, TRANS4		
	(And possible inter-occasion variability on clearance, central volume, and first inter-compartment clearance)		
			Discrete: sex
Escitalopram, oral [21]	ADVAN2, TRANS2	FOCE with interaction	Continuous: age, body-mass index, weight
	ADVAN4, TRANS4		Discrete: clinical site, CYP2C19 genotype, race, sex
Olanzapine, oral [14]	ADVAN2, TRANS2	FOCE with interaction	Continuous: age
	ADVAN4, TRANS4		Discrete: race, sex, smoking status
Perphenazine, oral [15]	ADVAN2, TRANS2	FOCE with interaction	Continuous: age, number of cigarettes per day, weight
	ADVAN4, TRANS4		
			Discrete: race, sex, smoking status, concomitant medication use
Risperidone, oral [16]	ADVAN2, TRANS2	FO	Continuous: age, weight
	ADVAN4, TRANS4		Discrete: race, sex, smoking status, concomitant medication use
	(And 1, 2, or 3 clearance subpopulations)		
Ziprasidone, oral [17]	ADVAN2, TRANS2	FOCE with interaction	Continuous: age, height, number of cigarettes per day, weight
			Discrete: race, sex, smoking status, concomitant medication use

^a^ADVAN2 is a one-compartment linear model with first order absorption, ADVAN3 is a two compartment linear model; ADVAN4 is a two compartment linear model with first order absorption; and ADVAN11 is a three compartment linear model

^b^TRANS2 with ADVAN2 specifies model options for clearance (CL) and volume of distribution (V); TRANS4 with ADVAN3 or ADVAN4 specifies model options for CL, central volume (V1), inter-compartment clearance (Q), and peripheral volume (V2); and TRANS4 with ADVAN11 specifies model options for CL, V1, first inter-compartment clearance (Q1), V2, second inter-compartment clearance (Q2), and third compartment volume (V3)

In addition to the structure, the initial values of the parameter estimates (and acceptable minimum and maximum values) must be specified. The SOHGA methods treats the combination of model structure and initial estimates as a single entity so multiple initial parameter values for the same model structure can be considered resulting in a search on initial estimates for parameters as well as the model structure.

### Functional forms of covariate relationships, inter-individual variability, and residual error variability for modeling clinical datasets

The stepwise method and SOHGA were each used to make decisions regarding model compartment structure, covariate inclusion as well as functional form of the covariate relationships, inter-individual variability, and residual error variability. Only function forms considered in the stepwise approach for modeling a compound were options for the SOHGA approach for that compound (see Table 4). Continuous covariate relationships included: no relationship, additive (add), proportional (prop), exponential (exp), power-law (pow), or Michaelis–Menten (MM). Discrete covariate relationships included: no relationship, additive, proportional, or exponential. The typical value of the pharmacokinetic model parameters were calculated by sequentially incorporating the effects of the individual covariate relationships for a patient with a given set of continuous and discrete covariates beginning with a covariate free typical value. The model variable value without covariates as well as the covariate function forms and corresponding parameters were included in the model fits to data. Covariates considered for inclusion by the model are listed in Table 3. All continuous covariates were centered at zero.

**Table 4:** Covariate, inter-individual variability, and residual error relationships considered during model building

	Mathematical function	Compounds tested
Covariate relationship^a^		
Continuous covariate		
No relationship	${\text{TVCL}}_{i} = {\text{TVCL}}_{i}$	Citalopram, DMAG, Escitalopram, Olanzapine, Perphenazine, Risperidone, Ziprasidone
Additive	${\text{TVCL}}_{i} = {\text{TVZ}}_{i} + \left( {x_{i,j} - \widetilde{x}_{j} } \right)\theta_{j}^{{({\text{add}})}}$	Citalopram, DMAG, Olanzapine, Perphenazine
Proportional	${\text{TVCL}}_{i} = {\text{TVCL}}_{i} \left( {1 + \theta_{j}^{{({\text{prop}})}} \frac{{x_{i,j} }}{{\widetilde{x}_{j} }}} \right)$	Citalopram, DMAG, Escitalopram, Olanzapine
Exponential	${\text{TVCL}}_{i}^{{}} = {\text{TVCL}}_{i}^{{}} {\text{exp}}\left( {\frac{{x_{i,j} }}{{\widetilde{x}_{j} }}\theta_{j}^{{({ \exp })}} } \right)$	Citalopram, DMAG, Escitalopram, Perphenazine, Risperidone, Olanzapine, Ziprasidone
Power-law	${\text{TVCL}}_{i}^{{}} = {\text{TVCL}}_{i}^{{}} \left( {\frac{{x_{i,j} }}{{\widetilde{x}_{j} }}} \right)^{{\theta_{j}^{{({\text{pow}})}} }}$	Citalopram, Escitalopram, Olanzapine, Perphenazine
Michaelis–Menten	${\text{TVCL}}_{i}^{{}} = {\text{TVCL}}_{i}^{{}} \frac{{\theta_{j,1}^{{({\text{MM}})}} \left( {\frac{{x_{i,j} }}{{\widetilde{x}_{j} }}} \right) }}{{\theta_{j,2}^{{({\text{MM}})}} + \left( {\frac{{x_{i,j} }}{{\widetilde{x}_{j} }}} \right) }}$	DMAG
Discrete		
No relationship	${\text{TVCL}}_{i}^{{}} = {\text{TVCL}}_{i}^{{}}$	Citalopram, DMAG, Escitalopram, Olanzapine, Perphenazine, Risperidone, Ziprasidone
Additive	${\text{TVCL}}_{i}^{{}} = {\text{TVCL}}_{i}^{{}} + \mathop \sum \limits_{k = 2} \delta \left( {k - x_{j|i} } \right)\theta_{j,k}^{{({\text{add}})}}$	Citalopram, DMAG, Escitalopram, Olanzapine, Perphenazine, Ziprasidone
Proportional	${\text{TVCL}}_{i}^{{}} = {\text{TVCL}}_{i}^{{}} \left( {1 + \mathop \sum \limits_{k = 2} \delta \left( {k - x_{j|i} } \right)\theta_{j,k}^{{({\text{prop}})}} } \right)$	Perphenazine
Exponential	${\text{TVCL}}_{i}^{{}} = {\text{TVCL}}_{i}^{{}} {\text{exp}}\left( {\mathop \sum \limits_{k = 2} \delta \left( {k - x_{j|i} } \right)\theta_{j,k}^{{({ \exp })}} } \right)$	Risperidone, Perphenazine
Type of inter-individual variability^b^		
No relationship	${\text{CL}}_{i} = {\text{TVCL}}_{i}$	Citalopram, DMAG, Escitalopram, Olanzapine, Perphenazine, Risperidone, Ziprasidone
Exponential	${\text{CL}}_{i} = {\text{TVCL}}_{i,} \exp \left( {\eta_{i}^{(CL)} } \right)$	Citalopram, DMAG,Escitalopram, Olanzapine, Perphenazine, Risperidone, Ziprasidone
Type of residual variability^c^		
Additive	$Y_{i,j} = F_{i,j} + \varepsilon_{i,j}^{(1)}$	Citalopram, DMAG, Escitalopram, Olanzapine, Perphenazine, Risperidone, Ziprasidone
Proportional	$Y_{i,j} = F_{i,j} + \left( {1 + \varepsilon_{i,j}^{(1)} } \right)$	Citalopram, DMAG, Escitalopram, Olanzapine, Perphenazine, Risperidone, Ziprasidone
Combined additive and proportional	$Y_{i,j} = F_{i,j} \left( {1 + \varepsilon_{i,j}^{(1)} } \right) + \varepsilon_{i,j}^{(2)}$	Citalopram, DMAG, Escitalopram, Olanzapine, Perphenazine, Risperidone, Ziprasidone

^a^Modification of a typical value for the example of clearance of the *i*th individual by the *j*th covariate or where *x*_*i,j*_ is the value of the *j*th covariate for the *i*th patient, $\widetilde{x}_{j}$ is the median value of the *j*th covariate, $x_{j|i}$ is the category (counting) number of the *j*th covariate for the *i*th patient, and $\theta_{j}^{(n)}$ is a parameter for functional form *n* that describe the relationship of the *j*th covariate

^b^Inter-individual function forms considered for the example of the clearance of the *i*th individual. The $\eta^{{\left( {\text{CL}} \right)}}$ takes a standard normal distribution with standard deviation $\omega^{{({\text{CL}})}}$

^c^Residual variability function forms considered. The variable *Y*_*i,j*_ is the *j*th observation for the *i*th patient, *F*_*i,j*_ is the corresponding model prediction, and $\varepsilon_{i,j}^{(1)}$ and $\varepsilon_{i,j}^{(2)}$ take standard normal distributions with variances $\sigma_{1}^{2}$ and $\sigma_{2}^{2}$, respectively

Two inter-individual variability function forms were considered: no inter-individual variability and exponential (see Table 4). These functions operated on random effects which were assumed to follow normal distributions centered at zero with the standard deviations included in the model fit to data.

Three residual variability function forms were considered: additive, proportional, and combined additive and proportional (see Table 4). These functions operated on random effects that were assumed to follow normal distributions centered at zero with variances included in the model fit to data.

### Model evaluation

Six measures were used for model evaluations and comparisons: the pharmacokinetic objective function value (OBV), the SOHGA fitness function, Akaike information criterion (AIC), Bayesian information criterion (BIC), mean prediction error (MPE), and root mean squared error (RMSE).

The pharmacokinetic objective function was used in the stepwise approach for covariate, inter-individual error, and residual error term inclusion/elimination. The pharmacokinetic objective function is calculated by NONMEM and is twice the negative log likelihood plus a fixed constant offset. Statistical differences in models were determined using Chi-squared distribution with one degree of freedom.

The SOHGA fitness function was used to compare candidate models in the genetic algorithm. The SOHGA fitness function was the summation of objective function value plus penalty terms for decreased parsimony (separate terms for fixed effect terms or “THETA”s, inter-individual variability terms or “OMEGA”s, and residual error terms or “SIGMA”s), lack of convergence, failing covariance step, and failing the correlation test.

The AIC and BIC were used to compare the fitness of models obtained using the stepwise approach with those of the genetic algorithms. The ${\text{AIC}} = 2k - {\text{OBV}}$ and ${\text{BIC}} = k { \log }\left( n \right) + {\text{OBV}}$ where *k* is the number of model parameters and *n* is the number of data points.

The mean prediction error for each compound is

$$ {\text{MPE}} = \frac{1}{N}\mathop \sum \limits_{i = 1}^{N} \left( {{\text{IPRED}}_{i} - {\text{DV}}_{i} } \right) $$

and the root mean squared error is

$$ {\text{RMSE}} = \sqrt {\frac{1}{N}\mathop \sum \limits_{i = 1}^{N} \left( {{\text{IPRED}}_{i} - {\text{DV}}_{i} } \right)^{2} } , $$

where *N* is the total number of concentration measurements, DV_*i*_ is the measured concentration, and IPRED_*i*_ is the individual concentration prediction for the *i*th measurement.

### Determination of outcomes

The primary outcome, comparing the quality of best pharmacokinetic model fits for the stepwise and single-objective hybrid genetic algorithm methods to clinical data, was quantified by the difference in AIC between the stepwise and single-objective, hybrid genetic algorithm approaches. The prediction error was also assessed by the median difference in the absolute MPE and RMSE between final stepwise models and best SOHGA candidates. In a secondary analysis, model compartment structure and the success of the model convergence and covariance steps was determined from the NONMEM control and output files, respectively. In another secondary analysis, the agreement in including covariates and variability terms on model parameters between the final stepwise model and the SOHGA candidate model with the lowest AIC was measured using a kappa analysis (a statistical measure of inter-method agreement for classification) [24]. The median percent difference in the model parameter values between the stepwise and SOHGA models was calculated, for variables common between the two models, using the absolute differences with the stepwise model variable values as the reference.

## Results

### Models for simulated dataset

As shown in Table 5, all automated SCM, Lasso, SOHGA options examined correctly identified that body-mass index and creatinine clearance influence clearance. All automated SCM and SOHGA options correctly identified the gender effect on central volume but this covariate was not identified by Lasso. All automated SCM, Lasso, and SOHGA options failed to identify the effect of body surface area on central volume. All automated SCM options identified spurious height and an unrelated covariate effect on the volume of distribution. In addition, the SCM option with the less stringent backwards elimination criteria (*p* = 0.05 vs. 0.01 for other automated SCM options) identified a spurious weight covariate effect on clearance. No spurious covariate effects were identified by any of the Lasso method options. The SOHGA option with the 3.84 point penalty identified 3 spurious covariates and 1 of these spurious covariates was also identified by the SOHGA model with a 10 point penalty per parameter.

**Table 5:** True and spurious covariate relationships identified in the simulated data by the automated stepwise covariate modeling, Lasso, and SOHGA approaches and the models fit characteristics

Method	“True” covariates		Spurious covariates		Objective function value
	Clearance	Volume of distribution	Clearance	Volume of distribution	
Original model	BMI, CRCL	BSA, Sex	–	–	6101.2
Stepwise covariate modeling (SCM): *p* value for inclusion, *p* value for elimination					
0.05, 0.05	BMI, CRCL	Sex	WT	HT, CV1	6085.9
0.05, 0.01	BMI, CRCL	Sex	–	HT, CV1	6091.1
0.10, 0.01	BMI, CRCL	Sex	–	HT, CV1	6091.1
Lasso model	BMI, CRCL	–	–	–	6254.2
Single-objective, hybrid genetic algorithm					
3.84 point penalty per parameter	BMI, CRCL	Sex	BSA	HT, CV1	6086.7
10 point penalty per parameter	BMI, CRCL	Sex	–	HT	6097.9

*BMI* body mass index, *BSA* body surface area, *CRCL* creatinine clearance, *CV1* unrelated covariate 1, *HT* height, *WT* weight

As shown in Table 6, the typical values of clearance for the SCM (0.01, 0.01), unconstrained Lasso, and SOHGA with a 10 point per covariate penalty were 0.614, 0.704, and 0.672 L/h while the true value was 0.763 L/h and the typical values of volume of distribution were 1.66, 1.87, and 2.17 while the true value was 1.94 L.

**Table 6:** Effects of covariates on clearance and volume of distribution

Method	Typical parameter value (relative standard error)		Inter-individual variability (relative standard error)		Residual error	
	Clearance (L/h) – TVCL (%)	Volume of distribution (L) – TVV (%)	Clearance (L/h) – TVCL (%)	Volume of distribution (L) – TVV (%)	Additive component (%)	Proportional component (%)
True model	0.763	1.94	20.0 %	20.0 %	0.001	0.01
Automated stepwise covariate modeling: *p* value for inclusion, *p* value for elimination						
0.05, 0.05	0.612 (3.1)	1.64 (5.7)	16.7 (10.3)	23.4 (9.5)	6.32E-4 (15.4)	0.0944 (4.2)
0.05, 0.01	0.614 (3.1)	1.66 (5.6)	17.1 (10.4)	23.4 (9.5)	6.32E-4 (15.6)	0.0944 (4.2)
0.10, 0.01	0.614 (3.1)	1.66 (5.6)	17.1 (10.4)	23.4 (9.5)	6.32E-4 (15.6)	0.0944 (4.2)
Lasso model^a^	0.662 (NA)	1.88 (NA)	32.5 (NA)	30.6 (NA)	6.29E-4 (NA)	0.0946 (NA)
Single-objective, hybrid genetic algorithm						
3.84 point penalty per parameter	0.681 (3.1)	2.21 (5.0)	16.7 (10.0)	23.6 (9.4)	6.31E-4 (15.4)	0.0944 (4.2)
10 point penalty per parameter	0.672 (3.1)	2.17 (5.3)	17.1 (10.4)	24.2 (9.1)	6.32E-4 (15.5)	0.0944 (4.2)

^a^Relative standard errors denoted by “NA” are not available because the covariance matrix did not converge

### Models for clinical datasets, primary outcome—Akaike information criterion

The AIC value did not increase (or worsen) by more than 10 points for the hybrid genetic algorithm model versus the stepwise model for all 7 compounds (see Table 7). Of these, there were greater than 10 point improvements in the AIC with the hybrid genetic algorithm versus the stepwise approach for four compounds—citalopram (−22.3), DMAG (−22.3), olanzapine (−470.5), and risperidone (−278.1)—and minimal AIC changes for escitalopram (−0.1), perphenazine (−4.8), and ziprasidone (−4.5).

**Table 7:** Values of the Akaike information criterion (AIC), Bayesian information criterion (BIC), and pharmacokinetic objective function value (OBV) for the final stepwise model and the single-objective, hybrid, genetic algorithm (SOHGA) candidate model with the lowest fitness function value

Compound	Final stepwise model	Final SOHGA model	AIC_SOHGA_ − AIC_stepwise_
Citalopram, IV	BIC = 5,474.9	BIC = 5,457.8	−22.3
	AIC = 5, 391.9	AIC = 5,369.6	
	OBV = 5,359.9	OBV = 5,335.6	
DMAG, IV	BIC = 9,942.3	BIC = 9,920.0	−22.3
	AIC = 9,871.7	AIC = 9,849.4	
	OBV = 9,843.7	−OBV = 9,821.4	
Escitalopram [21]	BIC = 2,800.1	BIC = 2,783.4	−0.1
	AIC = 2,737.7	AIC = 2,737.6	
	OBV = 2,707.1	OBV = 2,715.6	
Olanzapine, oral [14]	BIC = 10,413.8	BIC = 9,937.9	−470.5
	AIC = 10,365.8	AIC = 9,895.3	
	OBV = 10,347.8	OBV = 9,879.3	
Perphenazine, oral [15]	BIC = 601.1	BIC = 604.4	−4.8
	AIC = 560.7	AIC = 555.9	
	OBV = 540.7	OBV = 531.9	
Risperidone, oral [16]	BIC = 5,202.8	BIC = 4,934.9	−278.1
	AIC = 5,131.1	AIC = 4,853.0	
	OBV = 5,103.1	OBV = 4,821.0	
Ziprasidone, oral [17]	BIC = 4,789.3	BIC = 4,784.8	−4.5
	AIC = 4,763.2	AIC = 4,758.7	
	OBV = 4,751.2	OBV = 4,746.7	

### Models for clinical datasets, Secondary outcomes—bias and precision

The RMSE of the best SOHGA candidate was a median of 0.2 points higher (worse) than that of the final stepwise model (range of 38.9 point decrease to 27.3 point increase). As shown in Table 8, the RMSE of the best SOHGA candidate was greater than 10 points lower for citalopram (−38.9) but higher for DMAG (27.3) and olanzapine (21.7). The absolute value of the MPE of the best SOHGA candidate was a median of 0.02 point lower (improvement) than that of the final stepwise model (range of 0.98 point decrease to 0.74 point increase).

**Table 8:** Mean prediction error (MPE) and root mean square error (RMSE) for the final stepwise model and the single-objective, hybrid genetic algorithm (SOHGA) candidate model with lowest fitness function value

Compound	Mean prediction error (MPE)			Root mean square error (RMSE)		
	Final stepwise model	Best SOHGA candidate model	abs(MPE_SOHGA_) − abs(MPE_stepwise_)	Final stepwise model	Best SOHGA candidate model	RMSE_SOHGA_ − RMSE_stepwise_
Citalopram	0.46	0.28	−0.18	92.2	53.3	−38.9
DMAG	−5.49	−4.51	−0.98	1,167.8	1,195.1	27.3
Escitalopram	−1.16	−1.06	−0.10	132.6	133.1	0.5
Olanzapine	0.52	−1.26	0.74	345.4	367.1	21.7
Perphenazine	−0.19	−0.17	−0.02	14.9	15.1	0.2
Risperidone	−0.34	0.81	0.47	116.9	116.5	−0.4
Ziprasidone	8.14	8.31	0.17	622.0	613.8	−8.2

### Models for clinical datasets, secondary outcomes—model structure, convergence and covariance steps, and parameters

The compartment structures between the final stepwise and best SOHGA candidate agreed for six of the seven (86 %) compounds with risperidone as the single exception (see Appendix B). For the risperidone dataset, the best SOHGA candidate used a two compartment model (ADVAN4) whereas the final stepwise model used a one compartment model (ADVAN2). Post hoc assessment of the best SOHGA candidate (ADVAN4) versus the same model with ADVAN2 confirmed, by the likelihood ratio test, that the two additional parameters (k23 and k32) in the best SOHGA candidate were statistically significant (∆OBV = 146) at *p* < 0.01.

The best SOHGA candidate model had successful convergence and covariance steps for each compound (Table 9). All final stepwise models also converged, although the absorption rate constant (*K*_a_) was fixed for four compounds due to aid the convergence; the covariance step was unsuccessful for two compounds.

**Table 9:** Completion of convergence and covariance steps for the final stepwise model and the single-objective, hybrid genetic algorithm (SOHGA) candidate model with lowest fitness function value

	Convergence		Covariance step (condition number)	
	Final manual stepwise model	Best SOHGA candidate	Final stepwise model	Best SOHGA candidate
Citalopram, IV	Successful	Successful	Unsuccessful (N/A)	Successful (2,830)
DMAG, IV	Successful	Successful	Successful (20)	Successful (25)
Escitalopram [21]	Successful	Successful	Successful (39)	Successful (9)
Olanzapine, oral [14]	Required fixing *K*_a_ early in model building process	Successful	Successful (12)	Successful (50)
Perphenazine, oral [15]	Required fixing *K*_a_ early in model building process	Successful	Unsuccessful (N/A)	Successful (212)
Risperidone, oral [16]	Required fixing *K*_a_ early in model building process	Successful	Successful (61)	Successful (1.17x10^6^)
Ziprasidone, oral [17]	Required fixing *K*_a_ early in model building process	Successful	Successful (3)	Successful (5)

Comparison of the inclusion of covariates between the final stepwise and best SOHGA models had a kappa value of 0.32 which implies fair agreement of the covariate recognition (see Appendix B). The hybrid GA models included 54 % (7 of 13) of covariates in the stepwise models and the stepwise model included 30 % (7 of 23) of covariates in the best-fitting candidate SOHGA models.

The inclusion of inter-individual and inter-occasion variability between the final stepwise and hybrid SOHGA models had a kappa value of 0.28 which implies fair agreement. For stepwise model parameters with variability, the SOHGA also included variability terms on 64 % (14 of 21) of the parameters while the stepwise models included variability on 93 % (14 of 15) of parameters with these terms in the best SOHGA candidate models.

The median percent difference in parameter values (see Appendix C) between the final stepwise and best SOHGA candidate models for a compound was 26.7 % (inter-quartile range of 4.9–57.1 %).

### Models for clinical dataset, Secondary analysis—best SOHGA candidate model using FOCE with interaction for the risperidone dataset

The SOHGA approach identified a best candidate model that successfully converged for risperidone dataset using FOCE with interaction (OBV = 4,738.7 and condition number = 38). The best SOHGA candidate with FOCE used a two compartment model with absorption (ADVAN2/TRANS2) and a two compartment mixture for clearance. The best SOHGA candidate using FOCE with interaction included covariate effects of age, fluoxetine use, sex, and weight on clearance and inter-individual variability of clearance and the volume of distribution.

Comparing the structure of models developed using FOCE with interaction versus those using FO, the best SOHGA candidate using the FOCE with interaction method implemented the same compartment structure as the final stepwise model using FO (one compartment) but a different compartment structure than the best SOHGA candidate using FO (two compartment). In addition, the mixture model of the best SOHGA candidate using FOCE with interaction (two components) is different from that the final models that used FO (three components).

### Models for clinical datasets, post hoc analysis—best SOHGA candidate models with condition number penalty

Without a condition number penalty term in the fitness function, the best SOHGA candidate had a condition number >1,000 for two compounds (see Table 9): citalopram (condition number = 2,830) and risperidone (condition number = 1.17 × 10^6^). With a condition number penalty term included in the SOHGA analysis, the condition numbers for the best candidate models were 426 and 134 for citalopram and risperidone, respectively. Both of the post hoc best SOHGA candidates used the same compartment structure as the best models from the planned analyses.

With a condition number penalty included in the SOHGA analysis of the citalopram dataset, the AIC for the best SOHGA candidate (5,389.6) was 2.3 points lower than that of the final stepwise model and 20.0 points higher than that of the best SOHGA candidate without a condition number penalty. The best post hoc SOHGA candidate for citalopram had a similar MPE (−0.19) to the final stepwise model but the RMSE (52.2) was 40 points lower than that of the final stepwise model. The best post hoc, SOHGA candidate included covariate effects of sex on the central volume, weight on the inter-compartment clearance, and weight on the peripheral volume. This candidate also included inter-individual variability terms on central volume, inter-compartment clearance, and peripheral volume.

With a condition number penalty included in the SOHGA analysis of the risperidone dataset, the AIC for the best SOHGA candidate (4,855.1) was 275.4 points lower than that of the final stepwise model and 2.7 points higher than that of the best SOHGA candidate without a condition number penalty. The best post hoc SOHGA candidate for risperidone had a similar MPE (−0.36) to the final stepwise model but the RMSE (125.8) was 9.9 points higher than that of the final stepwise model. The best post hoc, SOHGA candidate used a three compartment mixture for clearance and included covariate effects of age, paroxetine use, and sex on clearance. This candidate also included inter-individual variability terms on clearance, central volume, and inter-compartment clearance.

## Discussion

We hypothesized that a single-objective, hybrid genetic algorithm (SOHGA) approach for selection of covariates and function forms in pharmacokinetic model building would accurately identify covariates, models, and appropriate initial estimates of model parameters with equal or superior fits to clinical data versus a manual stepwise method building approach. For covariate identification in a simulated dataset, we found that a SOHGA with a 10 point penalty per covariate correctly identified as many true covariates as an automated stepwise covariate modeling (three of four covariates) but with fewer spurious covariate relationships (1 vs. 2 spurious covariates). When applied to clinical datasets, we found that the best SOHGA candidate models outperformed the final manual stepwise models by at least 10 points in the AIC for four of seven compounds with nominal differences for three compounds and 10 point worse performance for none of the compounds. These results are consistent with the finding of a previous single compound pilot study in which the genetic algorithm approach to pharmacokinetic model building resulted in a better fit to clinical data than a stepwise modeling approach [3].

For the fits to simulated data, as expected, models with more stringent criteria for covariate incorporation had fewer spurious covariates. Changing the *p* value for exclusion from 0.05 to 0.01 in the automated SCM method led to 1 fewer spurious covariates and increasing the point penalty in the SOHGA from 3.84 to 10 points per covariate led to 2 fewer spurious covariates. While it is possible that a more stringent criteria for covariate incorporation could lead to exclusion of true covariates, this was not this case in this dataset as all automated SCM and SOHGA options identified three of four true covariates. However, all automated SCM and SOHGA options failed to identify the effect of body surface area on the volume of distribution. This was unexpected because the change in volume due to body surface area was expected to be equivalent to the change due to gender and gender was identified as a significant covariate by all automated approaches. It is possible that individual volumes of distribution happened to be selected from the random distribution such that the effect of body surface area was diminished from the expected value.

The typical values of parameters estimated by the automated SCM, Lasso, and SOHGA methods were all similar to those of the true model. This suggests that the SOHGA can accurately identify parameter values. However, this results is not generalizable based on a single simulation study and does not imply that SOHGA parameter estimates will be accurate for all possible datasets and model scenarios.

It is important to note that, although both the SCM and SOHGA included the spurious covariate of height as a predictor of volume rather than the true covariate of body-surface area, the objective function values for these models are lower than for the true model (3.3 points lower for SOHGA and 10.1 points lower for SCM). This suggests that, based on the change in objective function, both methods selected the better covariate although it was not the true (used in the simulation model) covariate. The correlation between height and body-surface area was high (0.92) so the effect of height was likely due to its correlation with body-surface area.

For the clinical datasets, the best SOHGA candidate models of all seven compounds successfully converged while the stepwise approach fixed the absorption rate constant for four compounds to achieve convergence. In addition, the SOHGA approach identified a candidate model for the risperidone which successfully converged using the FOCE with interaction approach which the stepwise approach did not. This suggests that, the SOHGA approach identified regions of solution space that converged in which the stepwise approach did not. This is most likely because the SOHGA approach can search the error, covariate, and initial estimate space for regions that converge and select candidate models from within those regions. Fixing the absorption rate constant in the stepwise approach restricted the models to a particular region of solution space. The improvements in AIC with the SOHGA approach to modeling olanzapine (∆AIC = −468.5) is likely due to the extra degree of modeling freedom provided by the absorption rate model parameter which the stepwise approach fixed based on literature values. This parameter was fixed early in the manual model building process due to a failure of the model to converge and was not revisited after subsequent changes were made to the model. This inability to estimate a specific parameter at one point in the modeling process but have the parameter be identifiable when other features are present is an example of the interdependence of model features and is similar to the documented interaction between compartments, variance terms, and covariates [2]. In contrast, the SOHGA method revisits and retests such a “decision” multiple times and in different combinations with other model features. This reduces the risk of missing important features as well as making it more likely to select the numerically optimal combination of features.

The lower AIC with the SOHGA approach to modeling citalopram (∆AIC = −22.3) is most likely due to the inclusion of additional covariate terms in the models. The best SOHGA candidate model for citalopram captures two of the three covariates of the stepwise model along with four additional covariate relationships and nearly identical the error structures between the two models. These model improvements suggest that the SOHGA identified interactions between model components that were not recognized by the stepwise approach. The lower AIC for risperidone with the SOHGA (∆AIC = −278.1) is likely a combination variable absorption rate and additional covariates; the best SOHGA candidate model for risperidone included four covariate terms versus zero for the final stepwise model although the best SOHGA model has one less mixture compartment for clearance. Finally, the lower AIC for DMAG with the SOHGA (∆AIC = −22.3) is most likely due to application of inter-occasion variability on clearance as opposed to the central volume.

However, the minor differences RMSE between the final stepwise models and best SOHGA candidates suggests that the improvements seen in AIC and the model description of data may not necessarily translate to benefits in predictive value.

Although a condition number penalty was not included in the SOHGA fitness function, the best SOHGA candidate model had condition numbers <1,000 for all compounds except for citalopram and risperidone. This suggests that the data was capable of supporting the best SOHGA candidate models except for those of citalopram and risperidone. However, with the addition of the condition number penalty, the SOHGA approach identified candidate models for citalopram and risperidone with condition numbers <1,000.

Over the seven compounds, the best SOHGA candidate models included more covariates than the stepwise approach (23 vs. 13) while including fewer variability terms (15 vs. 22); the SOHGA tended to describe variability with covariates rather than unknown variability relative to the stepwise approach. This was despite the penalty for fixed effect (THETA) and inter-individual variability (OMEGA) terms being equal (10 points). However, the best SOHGA candidate models did not include half of the covariates identified by the stepwise approach. This raises concerns about whether these covariates are an artifact of the model building approach [2].

It should be noted that the criteria for inclusion of a covariate in the stepwise regression was 3.84, based on the likelihood ratio test while the criteria for inclusion in the SOHGA approach was 10 points. This higher threshold for inclusion of a covariate effect in the SOHGA may be responsible for the lack of inclusion of other covariate relationships that were found using the stepwise regression approach. However, as demonstrated in the simulated dataset, this higher threshold for inclusion also provides some protection from inclusion of spurious covariates.

The 26.7 % median change in pharmacokinetic model parameters between the final stepwise and best SOHGA candidate models suggests that the model parameters are generally comparable. Much of this difference may be due to the fixed absorption rate constants in the final stepwise models and the differing covariate, variability, and error structures between the final stepwise and best SOHGA candidate models.

The strengths of this study are that the SOHGA method was tested on multiple compounds with data coming from multiple sites. The SOHGA was also applied over various conditions as the data for different compounds covered various time scales (months for SZ patients, weeks for AD patients, and hours for citalopram and DMAG), both oral and intravenous administration, and degrees of data and patient sample sizes and sparseness.

This study has several limitations. First, all models were either one-, two-, or three-compartment models, so the generalizability of the effectiveness of SOHGA approach to other model structures is uncertain. However, the SOHGA approach is entirely general and can be used for models described by ordinary differential equations, mixture models (such as the mixture model on clearance of risperidone implemented in this study), and odd type data. Another limitation is that the study involved mostly schizophrenia and Alzheimer’s patients and medications. The extent to which these results are generalizable to other medications and patients is uncertain.

There are also general limitations to using a genetic algorithm. While genetic algorithms quickly converge to “good” regions of the solution space, convergence to the “best” local and global solutions is much slower due to the random nature of the method. The hybrid technique aids in identifying local optima but convergence to the global optimum within the simulation cannot be guaranteed. Another consequence is that the genetic algorithm approach could result in multiple, equally valid solutions from different regions of the solution space. If these regions have different characteristics, it would be difficult to draw conclusions about covariates and model structure from strictly numerical results. This emphasizes the important role for the modelers in assessing biological plausibility, graphic diagnostics, and clinical importance of model features. While genetic algorithms can identify globally optimal solutions, the robustness of these solutions was not considered here. Finally, genetic algorithms are most efficient when the candidate models are evaluated in parallel rather than series so multiple core computers are recommended. The SOHGA models presented here took 6–150 h on a 24 processor computer server. More computational power, as is readily available with cloud computing, grid computing, or other shared resources methods, would decrease the run times. However, given this modest hardware configuration and the weeks to months of time to develop a model using manual approaches, the computational costs are not overly burdensome.

Also, the use of a single objective function can potentially introduce biases due to the ad hoc weighting. In this analysis, a ten point penalty for each model parameter was chosen based on the “Sheiner criteria.” (Lewis Sheiner, personal communication with one of the authors (MS). Dr. Sheiner explained that he often used 10 points as his criteria to include a parameter to correct for multiple comparisons, and because he rarely could see any difference in plots if the change in OBV was less than 10 points.) A smaller penalty would likely lead to more covariates in the model (and better overall fit but with a higher chance of spurious covariates) and a higher penalty would lead to inclusion of fewer covariates (and worse overall fit but with a lower chance of spurious covariates). The large penalty assigned for failure of convergence and failure of the covariance step (and the condition number, when implemented) was to give a high priority to these outcomes, as some modelers feel strongly that a successful covariance step is a necessary (but not sufficient) condition for a “final” model. A potential solution to these ad hoc penalties is the use of a multi objective function—with a dimension for each penalty term in the single objective algorithm—would yield a front of non-dominated optimal solutions [9]. However, both of these approaches (single objective and multi objective optimization) to the covariance step rely on a pass/fail criterion. Information from the covariance step output can often be used to understand additional opportunities to improve the model. However, hypothesis generation is the responsibility of the user and additional hypotheses to improve the model can be used to expand the search space on subsequent SOHGA analyses or by traditional forward addition/backward elimination.

The SOHGA algorithm presented here also does not consider the feasibility of or prior knowledge about model parameters. However, these factors could be included by introducing additional weighting in the fitness function. That is, an additional bonus is applied for covariates that the modeler feels should be included (e.g., a weight effect on volume of distribution) and a penalty is imposed for undesired interactions (e.g., having both body-mass index and weight modify a variable). While this weighting cannot guarantee the inclusion/exclusion of model parameters, it does shift the likelihood based on the modelers input.

Finally, using the SOHGA approach may result in the temptation to inadequately explore model parameter relationships and potential bias using graphics in pharmacokinetic model building. That is, the user may be tempted to spend less time looking at diagnostic plots and generating biologically sound hypotheses. The SOHGA cannot replace thorough examination of the data and hypothesis generation; SOHGA only automates the actual search part of the stepwise regression (e.g., construction of control files, running the model, quantifying results and construction of new control files). All intellectual input into the model selection process—the examination of graphics and hypothesis generation—remains the responsibility of the modeler. In a manner similar to traditional stepwise analysis, the best models from a SOHGA analysis can be examined using traditional diagnostic plots or other means and additional hypotheses generated to explain observed bias. All models run by SOHGA are available and the interface makes in convenient to identify models with desirable characteristics such as lower value of the objective function or parsimony. The search space can then be expanded to include these new hypotheses before SOHGA is run again.

Further, these results do not suggest that SOHGA, or any automated search algorithm, is likely to provide the final model for an analysis. A more practical approach to arriving at the “final” model may be that SOHGA would provide something comparable to initial parameter estimates for non-linear regression. That is, it is common practice to start a model building exercise with a trivial model (e.g., one compartment, no covariates, no inter-occasion variability, no mixture models, etc.), but it is likely that, while probably not the final model, a model from SOHGA will be closer to the true global minimum in the search space than the traditional trivial model. Given what is known about the failure of the assumption of monotonicity of the model search space [2], having a better starting place in the search space may result in a higher likelihood of finding the global minimum using traditional forward addition and backward elimination model building methods rather than a local minimum. A likely use scenario might be that the user assesses a number of the “best” models selected by SOHGA (e.g., those with low OBV, the most parsimonious, etc.) and the strengths and biases of each model can be assessed using standard diagnostic graphics and methods as well as biological plausibility. The features from different models can be recombined based on these assessments. As is the case for non-linear regression, the number of iterations may be fewer if a better starting point is provided.

It is likely that, initially at least, the SOHGA approach is best suited for compounds in which the biology is fairly well understood, and not for highly exploratory analyses. There are two reasons for this. First, experience suggests that hypotheses in poorly understood compounds typically come in small numbers—often one at a time—after examination of plots. The SOHGA approach requires that many (although not all) hypotheses be available initially. As discussed above, it is reasonable to perform a SOHGA search, examine the results and conclude that additional hypotheses are required, and then perform another SOHGA search with an expanded search space. But, if the drug is very poorly understood, few hypotheses may be available prior to the start of the model building. As a result, a process of running SOHGA with only a few hypotheses, examining the results, and generating new hypotheses may become even more tedious than step wise regression. Second, SOHGA is very computationally intensive. Complex model often require ordinary differential equation solutions, which can be very computationally intensive as well, making SOHGA impractical.

In conclusion, our results suggest that a single-objective, hybrid genetic algorithm can be used to fit pharmacokinetic model structures and covariates to data. This approach could be used either stand-alone or to identify regions of the solution space that could be explored further manually. Further additions to the genetic algorithm could include other objective measures of model quality and multi-objective optimization. Genetic algorithms provide a systematic way to identify covariates, interactions, initial parameter estimates, and the model structure for pharmacokinetic models accounting for interactions among model components that may otherwise be difficult to identify.

### Electronic supplementary material

Below is the link to the electronic supplementary material.

Supplementary material 1 (DOCX 465 kb)

## Appendix

For Appendices A, B, and C, see Tables 10, 11, and 12, respectively.

**Table 10:** Correlation matrix of simulated covariates and the NONMEM control file used for generation of simulated patient dataset

	BMI	HT	CR	AGE	WT	BSA	CRCL	CV1	CV2	CV3	CV4
BMI	1.000										
HT	0.072	1.000									
CR	−0.025	−0.059	1.000								
AGE	−0.065	−0.059	0.086	1.000							
WT	0.571	−0.041	−0.070	−0.071	1.000						
BSA	0.441	0.923	−0.066	−0.063	0.985	1.000					
CRCL	0.476	0.794	−0.368	−0.255	0.916	0.906	1.000				
CV1	0.058	−0.078	0.072	−0.010	−0.023	−0.043	−0.050	1.000			
CV2	0.134	0.000	0.012	0.041	0.070	0.049	0.057	0.010	1.000		
CV3	−0.048	−0.061	0.029	−0.044	−0.073	−0.073	−0.071	0.019	0.000	1.000	
CV4	−0.008	−0.030	0.012	0.032	−0.026	−0.032	−0.048	0.024	0.020	−0.026	1.000

*BMI* body-mass index, *HT* height, *CR* creatinine, *AGE* patient age, *WT* weight, *BSA* body-surface area, *CRCL* creatinine clearance, *CV1* first unrelated covariate, *CV2* second unrelated covariate, *CV3* third unrelated covariate, *CV4* fourth unrelated covariate

**Table:**

$PROBLEM SIM MODEL
$INPUT ID TIME AMT DV BMI HT CR AGE GEN WT BSA GFR CV1 CV2 CV3 CV4
$DATA DATA.PRN
$SUBROUTINE ADVAN1 TRANS2
$PK
CBMI = (BMI-26.29)/4.093
CHT = (HT-1.586)/0.2
CCR = (CR-0.985)/0117
CAGE = (AGE-38.81)/8.189
CWT = (WT-67.4)/21.2
CBSA = (BSA-1.713)/0.363
CGFR = (GFR-91.4)/37.9
CCV1 = (CV1-101.6)/30.7
CCV2 = (CV2-11)/4.33
CCV3 = (CV3-1.01)/0.209
CCV4 = (CV4-0.098)/0.0198
TVCL = THETA(1)*EXP(THETA(2)*BMI)*EXP(THETA(3)*GFR)
CL = TVCL*EXP(ETA(1))
TVV = THETA(4)*EXP(THETA(5)*BSA)*EXP((1-GEN)*THETA(6))
V = TVV*EXP(ETA(2))
S1 = V
$ERROR
Y = F*EXP(EPS(1)) +EPS(2)
IPRED = F*EXP(EPS(1)) +EPS(2); individual-specific prediction
$THETA (0,0.1); BASELINE CL
$THETA (0,0.04); BMI ON CL
$THETA (0,0.01); crcl oncl
$THETA (0,1); baseline volume
$THETA (0,0.3); bsa on V
$THETA (0,0.3); GEN ON V
$OMEGA BLOCK(2)
.2
0.05 .2
$SIGMA 0.1 0.001
$SIMULATION (12345) ONLYSIM
$TABLE ID TIME AMT IPRED BMI HT CR AGE GEN WT BSA GFR CV1 CV2 CV3 CV4
NOPRINT FILE = OUT.DAT ONEHEADER

**Table 11:** Model structures and covariates of the final stepwise model and the single-objective, hybrid genetic algorithm (SOHGA) candidate model with the lowest fitness function value

Compounds	Covariates in stepwise model (abbreviation of function form^a^)	Covariates in the best SOHGA candidate model (abbreviation of function form^a^)
Citalopram, IV	ADVAN3, TRANS4 (2 compartments)	ADVAN3, TRANS4 (2 compartments)
Clearance—TVCL	Weight (pow)	–
Inter-individual variability—η^(CL)^	–	–
Central volume—TVV1	Sex (add)	Sex (exp), body-mass index (exp)
Inter-individual variability—η^(V1)^	Exponential	Exponential
Inter-comp clearance—TVQ	Fat mass (exp)	Fat mass (prop), fat-free mass (exp)
Inter-individual variability—η^(Q)^	Exponential	Exponential
Peripheral volume—TVV2	Weight (pow)	Fat (exp), fat-free mass (exp)
Inter-individual variability—η^(V2)^	Exponential	Exponential
Residual variability—ε^(1)^ and ε^(2)^	Combined	Proportional
DMAG, IV	ADVAN11, TRANS4 (3 compartments)	ADVAN11, TRANS4 (three compartments)
Clearance—TVCL	–	Sex (exp)
Inter-individual variability—η^(CL)^	Exponential	Exponential
Inter-occasion variability—η^(OCC,CL)^	–	Exponential
First comp clearance—TVQ1	–	–
Inter-individual variability—η^(Q1)^	–	–
Inter-occasion variability—η^(OCC,Q1)^	Exponential	Exponential
Second comp clearance—TVQ2	–	–
Inter-patient variability—η^(Q2)^	Exponential	–
Central volume—TVV1	–	–
Inter-individual variability—η^(V1)^	Exponential	Exponential
Inter-occasion variability—η^(OCC,V1)^	Exponential	–
Peripheral volume—TVV2	–	–
Inter-individual variability—η^(V2)^	Exponential	Exponential
Third volume—TVV3	–	–
Inter-patient variability—η^(V3)^	Exponential	–
Residual variability—ε^(1)^ and ε^(2)^	Proportional	Combined
Escitalopram, oral	ADVAN2, TRANS2 [17] (one compartment with absorption)	ADVAN2, TRANS2 (one compartment with absorption)
Clearance—TVCL	Age (pow), genotype (add), weight (pow)	Age (pow), site (add)
Inter-individual variability—η^(CL)^	Exponential	Exponential
Volume of distribution—TVV	Body mass index (pow)	Body mass index (pow)
Inter-individual variability—η^(V)^	Exponential	Exponential
Absorption rate constant—TVKA	–	–
Inter-individual variability—η^(KA)^	Exponential	–
Residual variability—ε^(1)^ and ε^(2)^	Proportional	Combined
Olanzapine, oral	ADVAN2, TRANS2 [10] (one compartment with absorption)	ADVAN2, TRANS2 (one compartment with absorption)
Clearance—TVCL	Race (add), sex (add), smoking status (add)	Age (add), sex (add)
Inter-individual variability—η^(CL)^	Exponential	Exponential
Volume of distribution—TVV	–	–
Inter-individual variability—η^(V)^	Exponential	–
Absorption rate constant—TVKA	Unstable: fixed based on literature	–
Inter-individual variability—η^(KA)^	–	–
Residual variability—ε^(1)^ and ε^(2)^	Additive	Combined
Perphenazine, oral	ADVAN2, TRANS2 [11] (one compartment with absorption)	ADVAN2, TRANS2 (one compartment with absorption)
Clearance—TVCL	Race (add), smoking status (add)	Fluoxetine use (exp), number of cigarettes per day (exp), race (exp), smoking status (prop)
Inter-individual variability—η^(CL)^	Exponential	Exponential
Volume of distribution—TVV	–	Age (exp), sex(exp), and smoking status (prop)
Inter-individual variability—η^(V)^	Exponential	–
Absorption rate—TVKA	Unstable: fixed based on literature	–
Inter-individual variability—η^(KA)^	Exponential	–
Residual variability—ε^(1)^ and ε^(2)^	Proportional	Proportional
Risperidone, oral	ADVAN2, TRANS2 with three component clearance mixture [12] (one compartment with absorption)	ADVAN4, TRANS4 with three component mixture on clearance (two compartment with absorption)
Clearance—TVCL	–	Age (exp), fluoxetine use (exp), paroxetine use (exp), race (exp)
Inter-individual variability—η^(CL)^	Exponential	Exponential
Volume of distribution—TVV	–	–
Inter-individual variability—η^(V)^	Exponential	–
Central volume—TVV1	–	–
Inter-individual variability—η^(V1)^	–	Exponential
Inter-comp clearance—TVQ	–	–
Inter-individual variability—η^(*Q*)^	–	–
Peripheral volume—TVV2	–	–
Inter-individual variability—η^(V2)^	–	–
Absorption rate constant—TVKA	Fixed based on literature	–
Inter-individual variability—η^(KA)^	Exponential	–
Residual variability—ε^(1)^ and ε^(2)^	Combined	Proportional
Ziprasidone, oral	ADVAN2, TRANS2 [13] (one compartment with absorption)	ADVAN2, TRANS2 (one compartment with absorption)
Clearance—TVCL	–	–
Inter-individual variability—η^(CL)^	Exponential	Exponential
Volume of distribution—TVV	–	–
Inter-individual variability—η^(V)^	Exponential	Exponential
Absorption rate constant—TVKA	Unstable: fixed based on literature	–
Inter-individual variability—η^(KA)^	–	–
Residual variability—ε^(1)^ and ε^(2)^	Proportional	Proportional

^a^Abbreviations for functional forms include: *add* additive, *prop* proportional, *exp* exponential, *pow* power-law

**Table 12:** Typical parameter values, inter-individual and inter-occasion variability, and residual error of the final stepwise model and the single-objective, hybrid genetic algorithm (SOHGA) candidate model with the lowest fitness function value

Compound	Parameter estimates^a,b,c^ (relative standard error)	
	Final stepwise model	Best SOHGA candidate model
Citalopram, IV—two compartment		
Clearance (L/h)—TVCL	7.11 (NA)	1.22 (5.3 %)
Inter-individual variability—ω_CL_ of η_CL_	–	–
Inter-comp clearance (L/h)—*TVQ*	64.4 (NA)	6.26 (19.0 %)
Inter-individual variability—ω_Q_ of η_Q_	48.5 % (NA)	35.5 % (19.0 %)
Central volume (L)—TVV1	2.87 (NA)	82.1 (17.7 %)
Inter-individual variability—ω_V1_ of η_V1_	12.2 % (NA)	46.2 % (15.5 %)
Peripheral volume (L)—TVV2	1,510 (NA)	205 (12.6 %)
Inter-individual variability—ω_V2_ of η_V2_	22.6 % (NA)	25.4 % (12.5 %)
Residual variability—σ of ε	σ_prop_ = 14.6 % (NA) and	σ_prop_ = 10.5 % (28.0 %)
	σ_add_ = 0.0 μg/L (NA)	–
DMAG—three compartment		
Clearance (L/h)—TVCL	8.4 (11.2 %)	9.28 (7.9 %)
Inter-patient variability—ω_CL_ of η_CL_	53.8 % (22.9 %)	22.1 % (29.1 %)
Inter-occasion variability—ω_CL_ of η_OCC,CL_	–	8.3 % (38.6 %)
First inter-compartment clearance (L/h)—TVQ1	85.1 (9.6 %)	81.0 (8.9 %)
Inter-patient variability—ω_Q1_ of η_Q1_	–	–
Inter-occasion variability—ω_Q1_ of η_OCC,Q1_	32.6 % (37.2 %)	14.0 % (32.7 %)
Second inter-compartment clearance (L/h)—TVQ2	11.6 (13.1 %)	8.21 (17.4 %)
Inter-patient variability—ω_Q2_ of η_Q2_	75.8 % (32.0 %)	–
First compartment volume (L)—TVV1	27.4 (11.7 %)	28.1 (12.9 %)
Inter-patient variability—ω_V1_ of η_V1_	33.0 % (111.9 %)	39.7 % (24.5 %)
Inter-occasion variability	59.7 % (31.2 %)	–
Second compartment volume (L)—TVV2	66.4 (10.1 %)	77.9 (11.4 %)
Inter-patient variability—ω_V2_ of η_V2_	50.7 % (23.7 %)	42.3 % (24.8 %)
Third compartment volume (L)—TVV3	142 (13.5 %)	86.4 (8.5 %)
Inter-patient variability—ω_V3_ of η_V3_	67.5 % (37.3 %)	–
Residual variability—σ of ε	σ_prop_ = 16.1 % (2.7 %)	σ_prop_ = 2.1 % (13.2 %) and
	–	σ_add_ = 24.7 μg/L (69.6 %)
Escitalopram, oral—one compartment with absorption		
Clearance (L/h)—TVCL	–	25.5 (5.3 %)
Extensive metabolizer	26 (7.20 %)	–
Poor metabolizer	19.8 (8.50 %)	–
Missing metabolizer information	21.5 (7.80 %)	–
Inter-individual variability—ω_CL_ of η_CL_	48.5 % (15.1 %)	48.8 % (12.3 %)
Volume of distribution (L)—TVV	947 (10.2 %)	874 (11.8 %)
Inter-individual variability—ω_*V*_ of η_*V*_	62.0 % (40.3 %)	59.4 % (32.6 %)
Absorption (1/h)—TVKA	0.8 (NA)	0.594 (23.1 %)
Inter-individual variability—ω_KA_ of η_KA_	78.9 % (87.0 %)	–
Residual variability—σ of ε	σ_prop_ = 28.9 % (8.8 %)	σ_prop_ = 28.8 % (11.1 %) and
	–	σ_add_ = 70.4 μg/L (131 %)
Olanzapine, oral—one compartment with absorption		
Clearance (L/h)—TVCL	16.1 (7.3 %)	21.1 (15.5 %)
Inter-individual variability—*ω*_CL_ of *η*_CL_	68 %	64.7 % (17.2 %)
Volume of distribution (L)—TVV	2,150 (26.0 %)	4,130 (48.9 %)
Inter-individual variability—ω_*V*_ of η_*V*_	86 %	–
Absorption rate constant (1/h)—TVKA	Fixed at 0.5	0.773 (70.4 %)
Inter-individual variability—ω_KA_ of η_KA_	–	–
Residual variability—σ of ε	–	σ_prop_ = 37.7 % (14.5 %) and σ_add_ = 275 μg/L (50.9 %)
	σ_add_ = 212 μg/L (1.7 %)	
Perphenazine, oral—one compartment with absorption	(95 % CI from bootstrapping)	
Clearance (L/h)—TVCL	483	354 (12.3 %)
Inter-individual variability—ω_CL_ of η_CL_	79.3 % (68.1 - 87.4 %)	78.8 % (11.4 %)
Volume of distribution (L)—TVV	18,200 (13,865 - 65,795)	1.10x10^7^ (172 %)
Inter-individual variability—ω_*V*_ of η_*V*_	78.5 % (20 - 120 %)	–
Absorption rate constant (1/h)—TVKA	Fixed at 1.6	0.859 (80.7 %)
Inter-individual variability—ω_KA_ of η_KA_	336.1 % (0.35 - 518 %)	–
Residual variability—σ of ε	σ_prop_ = 37.4 % (33.3 - 41.0 %)	σ_prop_ = 37.1 % (10.5 %)
Risperidone, oral—one compartment with absorption	3 component mixture for clearance	3 component mixture for clearance
Percent of subjects who were poor metabolizers	41.2 % (8.1 %)	22.4 % (4.4 %)
Percent of subjects who were extensive metabolizers	52.4 % (6.2 %)	41.4 % (8.6 %)
Clearance (L/h)—TVCL	–	–
Poor metabolizer	12.9 (6.5 %)	12.4 (8.5 %)
Intermediate metabolizer	Fixed at 36	34.9 (8.9 %)
Extensive metabolizer	65.4 (9.9 %)	122 (11.5 %)
Inter-individual variability—ω_CL_ of η_CL_	–	7.3 % (32.2 %)
Poor metabolizer	95.9 % (39.5 %)	–
Intermediate metabolizer	NA (NA)	–
Extensive metabolizer	56.6 % (16.8 %)	–
Volume of distribution (L)—TVV	444 (17.8 %)	–
Inter-individual variability—ω_*V*_ of η_*V*_	36.1 % (24.4 %)	–
Central volume—TVV1	–	96.6 (31.2 %)
Inter-individual variability—η^(V1)^	–	478 % (46.4 %)
Inter-comp clearance—TVQ	–	27.1 (29.9 %)
Inter-individual variability—η^(*Q*)^	–	–
Peripheral volume—TVV2	–	1.31x10^10^ (1,000 %)
Inter-individual variability—η^(V2)^	–	–
Absorption rate constant (1/h)—TVKA	Fixed at 1.7	0.184.(21.1 %)
Inter-individual variability—ω_KA_ of η_KA_	53.7 % (89.3 %)	–
Residual variability—σ of ε	σ_prop_ = 63.9 % (12.5 %) and	σ_prop_ = .39.8 % (9.3 %)
	σ_add_ = 4.29 μg/L (104.9 μg/L)	–
Ziprasidone, oral—one compartment with absorption		
Clearance (L/h)—TVCL	122 (9 %)	126 (9.0 %)
Inter-individual variability—ω_CL_ of η_CL_	64.8 % (25 %)	69.0 % (21.4 %)
Volume of distribution (L)—TVV	1,060 (19 %)	1,030 (12.4 %)
Inter-individual variability—ω_*V*_ of η_*V*_	104.4 % (27 %)	114 % (16.0 %)
Absorption rate constant (1/h)—TVKA	Fixed at 0.5	0.424 (5.8 %)
Inter-individual variability—ω_KA_ of η_KA_	–	–
Residual variability—σ of ε	σ_prop_ = 65.5 % (8 %)	σ_prop_ = 65.1 % (8.3 %)

^a^Parameter estimates denoted by “–” were not calculated and standard errors denoted by “NA” are not available because the covariance matrix did not converge

^b^The reported inter-individual variability value is the standard deviation (ω) of the random inter-individual variability variable (η), which is a normal distribution with near zero mean

^c^The reported residual variability value is the standard deviation (σ) of the residual variability variable (ε), which is a normal distribution with near zero mean
