# Supplementary material for: Application of a single-objective, hybrid genetic algorithm approach to pharmacokinetic model building
Source: J Pharmacokinet Pharmacodyn. 2012 Jul 6;39(4):393–414. doi: 10.1007/s10928-012-9258-0 (PMC3400037; doi:10.1007/s10928-012-9258-0)

**Supplemental Material - Numerical predictive distribution error (NDPE) plots for the final model fit to simulation data for each automated covariate identification method**

Original dataset


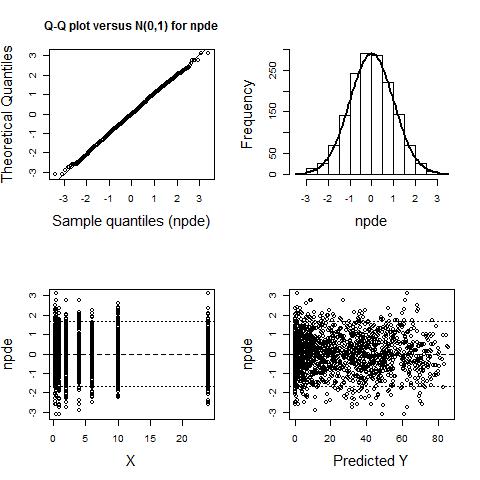


Automated stepwise covariate modeling: Forward addition P-value = 0.05 and backward elimination P-value = 0.05


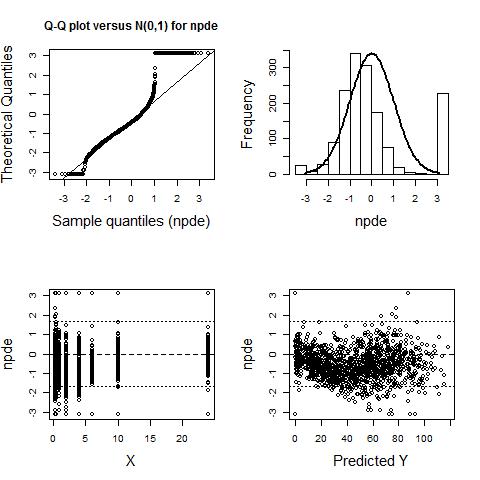


Automated stepwise covariate modeling: Forward addition P-value = 0.05 and backward elimination P-value = 0.01


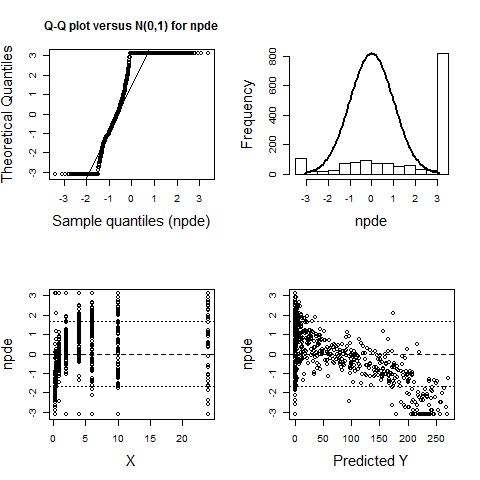


Automated stepwise covariate modeling: Forward addition P-value = 0.10 and backward elimination P-value = 0.01


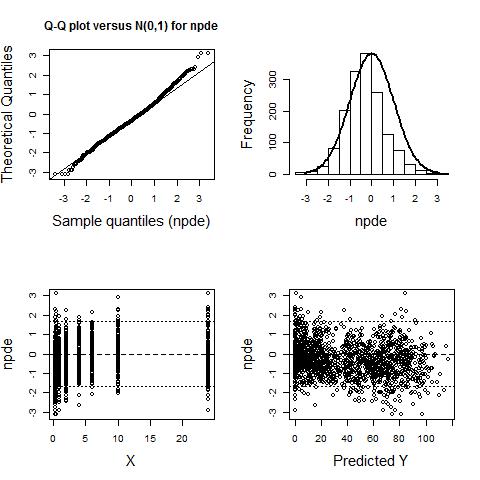


Lasso


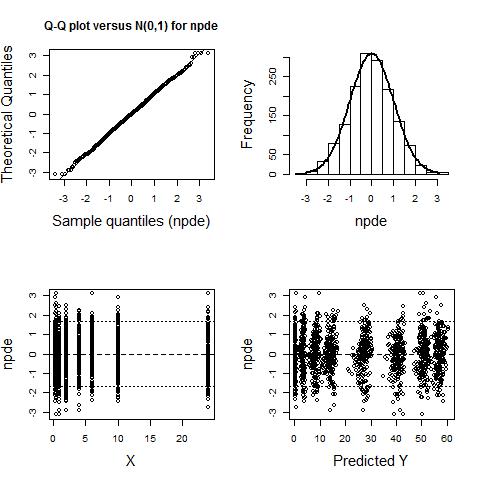


Lasso, uncontrained


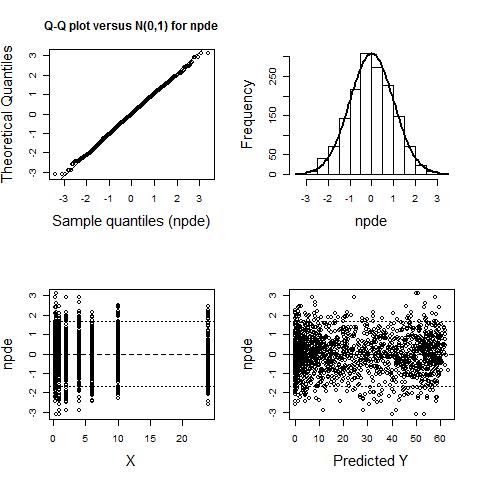


Single-objective, hybrid genetic algorithm with a 3.84 point penalty per covariate


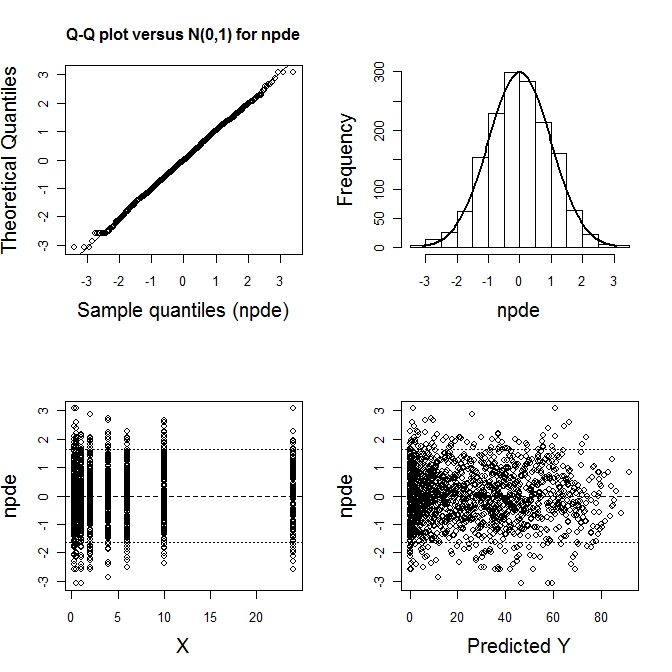


Single-objective, hybrid genetic algorithm with a 10 point penalty per covariate


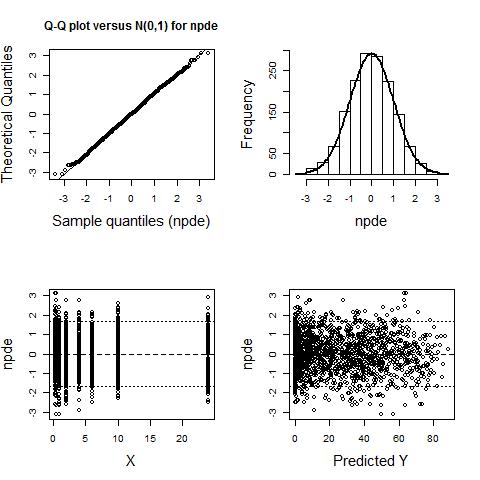

Supplement: Supplementary file 1 — Supplementary material 1 (DOCX 465 kb) [file 10928_2012_9258_MOESM1_ESM.docx]
